# Dietary Patterns, Micronutrient Adequacy, and Metabolic Vulnerability in Alzheimer’s Disease: A Critical Narrative Review

**DOI:** 10.3390/nu18183025

**Published:** 2026-09-16

**Authors:** Isidora Sierralta, María Pinilla, Evrim Servili, Paulina Ormazabal, Marianela Bastías-Pérez, Pedro Cisternas, Nibaldo C. Inestrosa

**Affiliations:** 1 Escuela de Salud, Universidad de O’Higgins, Rancagua 2820000, Chile; isidora.sierralta@pregrado.uoh.cl (I.S.); maria.pinilla@pregrado.uoh.cl (M.P.); servili.evrim@gmail.com (E.S.); 2 Facultad de Ciencias para el Cuidado de la Salud, Universidad San Sebastián, Lota 2465, Providencia, Santiago 7510157, Chile; paulina.ormazabal@uss.cl; 3 Centro de Investigación Biomédica en Red (CIBER) de Fisiopatología de la Obesidad y Nutrición (CIBEROBN), Instituto de Salud Carlos III (ISCIII), E-28029 Madrid, Spain; mbastias@udla.cl; 4 Núcleo de Investigación en Nutrición y Ciencias Alimentarias (NINCAL), Facultad de Salud y Ciencias Sociales, Universidad de Las Américas, Santiago 7500975, Chile; 5 Centro de Excelencia en Biomedicina de Magallanes (CEBIMA), Escuela de Medicina, Universidad de Magallanes, Punta Arenas 6210427, Chile; 6 Escuela de Nutrición y Dietética, Facultad de Salud y Ciencias Sociales, Universidad de Las Américas, Santiago 7500975, Chile

**Keywords:** Alzheimer’s disease, dietary patterns, Mediterranean diet, MIND diet, plant-based diet, ketogenic diet, vitamin B12, vitamin D, cognitive decline, nutritional status

## Abstract

Dietary factors may influence cognitive aging and Alzheimer’s disease (AD), but observational associations, mechanistic findings, and intervention effects should not be considered equivalent evidence. This critical narrative review examines how dietary patterns, micronutrient adequacy, and metabolic health may modify AD-related vulnerability. A targeted search of PubMed/MEDLINE, Scopus, and Web of Science through April 2026 prioritized systematic reviews, meta-analyses, randomized trials, and prospective cohorts. Mediterranean, Mediterranean–DASH Intervention for Neurodegenerative Delay (MIND), and healthful plant-based dietary patterns have been associated with better cognitive outcomes and lower dementia risk. However, evidence remains predominantly observational, and randomized trials have not consistently demonstrated prevention of cognitive decline. Strict vegan diets have not shown additional protection beyond high-quality plant-based diets and require careful attention to vitamin B12 and overall nutritional adequacy. Ketogenic diets and medium-chain triglycerides may increase cerebral ketone availability, but their cognitive benefits remain inconsistent, and disease modification has not been established. Although vitamin B12, folate, and vitamin D support neural and metabolic functions, correcting deficiency should not be equated with treating AD. Overall, nutrition should be viewed as a contributor to cardiometabolic and neurological resilience and as part of person-centered dementia care, rather than as a stand-alone disease-modifying therapy. Future studies should integrate accurate dietary assessment, objective adherence measures, metabolic phenotyping, AD biomarkers, and clinically meaningful outcomes.

## 1. Introduction

Alzheimer’s disease (AD) is a progressive neurodegenerative disorder and the most common cause of dementia, accounting for approximately 60–70% of cases [1,2]. More than 55 million people are estimated to be living with dementia worldwide, most of them in low- and middle-income countries [1]. This burden is expected to increase substantially as populations age, intensifying the clinical, social, and economic consequences for individuals, families, caregivers, and healthcare systems [3]. AD therefore represents both a neurological disorder and a major public health challenge requiring coordinated approaches to diagnosis, treatment, supportive care, and risk reduction.

Clinically, AD is characterized by progressive impairment of memory and other cognitive domains, followed by loss of functional independence. Its defining neuropathological features include extracellular amyloid-β (Aβ) deposition, intracellular accumulation of hyperphosphorylated tau in neurofibrillary tangles, synaptic dysfunction, and neuronal loss [4,5]. These features are central to the disease but do not fully explain the marked variation in age at onset, clinical expression, or rate of progression. Neuroinflammation, oxidative stress, mitochondrial dysfunction, vascular injury, and impaired cellular energy metabolism are increasingly recognized as interacting processes that may influence neuronal resilience and the transition from preclinical pathology to symptomatic disease [6,7].

Therapeutic options have expanded, but no available intervention cures AD or restores neuronal networks that have already been lost. Cholinesterase inhibitors and memantine provide symptomatic benefit in selected individuals, whereas anti-Aβ monoclonal antibodies such as lecanemab and donanemab have modestly slowed cognitive and functional decline in carefully selected patients with early symptomatic AD and confirmed amyloid pathology [8,9,10,11]. Their restricted eligibility, treatment-related risks, monitoring requirements, and limited effect size reinforce the importance of identifying modifiable factors that may preserve cognitive health or delay the clinical expression of disease.

AD develops through interactions among aging, genetic susceptibility, cardiovascular and metabolic health, environmental exposures, and lifestyle-related factors. Contemporary prevention frameworks emphasize that dementia risk accumulates over decades and may be influenced by modifiable conditions, including hypertension, diabetes, obesity, physical inactivity, smoking, depression, hearing loss, and social isolation [12,13].

Nutrition intersects with many of these factors through its effects on body composition, insulin sensitivity, lipid metabolism, blood pressure, vascular integrity, inflammation, and micronutrient availability. Its potential contribution should therefore be considered within a multidomain approach to cardiovascular, metabolic, and cognitive health rather than as an isolated intervention [14,15].

Metabolic dysfunction represents a particularly relevant link between diet and AD-related vulnerability. Metabolic syndrome and its individual components have been associated with cognitive impairment and dementia, although these relationships vary according to age, duration of exposure, and disease stage [16]. Reduced cerebral glucose metabolism can be detected in vulnerable brain regions before advanced clinical deterioration, while impaired insulin signaling, mitochondrial dysfunction, and reduced energy availability may compromise synaptic plasticity and neuronal resilience. Although the causal relationships linking peripheral metabolic disease, cerebral insulin resistance, and AD pathology remain incompletely resolved, they provide a biologically plausible framework for examining dietary exposures [17,18,19].

Mediterranean and Mediterranean–DASH Intervention for Neurodegenerative Delay (MIND) dietary patterns, plant-based and vegan diets, and ketogenic strategies have consequently attracted considerable attention. The MIND diet combines elements of the Mediterranean and Dietary Approaches to Stop Hypertension dietary patterns, while placing particular emphasis on foods proposed to support brain health, including green leafy vegetables, berries, nuts, whole grains, legumes, fish, poultry, and olive oil, and limiting red meat, butter, cheese, sweets, and fried or highly processed foods. These dietary approaches differ in food composition, metabolic objectives, restrictiveness, and risk of nutritional inadequacy [20,21,22]. Their neurological effects cannot be inferred from dietary labels alone, as food quality, energy balance, protein and fatty acid composition, micronutrient status, adherence, and individual characteristics must also be considered. Vitamin B12, folate, and vitamin D are especially relevant because their deficiencies may adversely affect neurological, vascular, and functional health; however, correcting a deficiency should not be equated with treating AD [23,24].

The relationship between nutrition and AD is also bidirectional. Poor dietary quality may contribute to metabolic and nutritional disturbances that increase vulnerability, whereas prodromal or established cognitive impairment can alter appetite, food preferences, meal preparation, mobility, sunlight exposure, and treatment adherence. Associations between dietary intake, circulating nutrient concentrations, and cognition should therefore not automatically be interpreted as causal. It is equally important to distinguish long-term associations with incident dementia from interventions initiated after mild cognitive impairment (MCI) or clinically established AD has developed. This critical narrative review examines how dietary patterns, micronutrient adequacy, and metabolic health may influence AD-related vulnerability. Particular attention is given to Mediterranean and MIND diets, plant-based and vegan patterns, ketogenic strategies, vitamin B12–folate–homocysteine metabolism, and vitamin D. The evidence is interpreted with emphasis on the distinctions between association and intervention, prevention and treatment, metabolic compensation and disease modification, and correction of nutritional deficiency versus supplementation in nutritionally sufficient individuals.

## 2. Literature Search Strategy

A targeted literature search was conducted in PubMed/MEDLINE, Scopus, and Web of Science from database inception through April 2026 to support this critical narrative review. Search terms related to AD and cognitive decline were combined with terms describing the dietary patterns, micronutrients, and metabolic pathways addressed in the manuscript. These included “Alzheimer’s disease,” “mild cognitive impairment,” “dementia,” “Mediterranean diet,” “MIND diet,” “plant-based diet,” “vegan diet,” “ketogenic diet,” “medium-chain triglycerides,” “vitamin B12,” “folate,” “homocysteine,” “vitamin D,” “brain glucose metabolism,” “insulin resistance,” “neuroinflammation,” and “oxidative stress.” Given the breadth of the nutrition–AD literature, the scope was intentionally focused on nutritional exposures that met one or more of three criteria: substantial human observational or interventional evidence relevant to cognitive aging or AD-related outcomes; direct clinical relevance to common nutritional or metabolic vulnerabilities in older adults; or a biologically plausible link to AD-related processes supported by human evidence. Mediterranean and MIND dietary patterns were prioritized because they have the most developed epidemiological and intervention literature in relation to cognitive outcomes. Plant-based and vegan patterns were included because of their increasing use, their potential cardiometabolic effects, and specific concerns regarding nutritional adequacy. Ketogenic and MCT-based strategies were selected because they directly address the reduction in cerebral glucose utilization observed across the AD continuum. Vitamin B12, folate, homocysteine, and vitamin D were included because deficiencies or altered status are common in older adults, clinically actionable, and potentially relevant to neurological and vascular health. The review was not intended to provide an exhaustive catalogue of all foods, nutrients, or supplements investigated in relation to dementia. To reduce the likelihood that major nutritional domains were overlooked, reference lists of recent reviews and key primary studies were cross-checked, and additional exposures were considered when they directly informed the selected themes.

Reference lists of relevant reviews and primary studies were also examined. Systematic reviews, meta-analyses, randomized clinical trials, and prospective cohorts were prioritized when available. Cross-sectional studies were considered when they provided relevant information on nutritional status or AD-related outcomes, whereas preclinical studies were included selectively to clarify mechanisms that could not be evaluated directly in humans. The literature search covered studies published from database inception through 30 April 2026. Publications from 2016 to 2026 were prioritized to reflect the most recent evidence, while studies published before 2016 were retained when they represented seminal clinical or mechanistic contributions. Articles published in peer-reviewed journals, either in their final version or online ahead of print by the cutoff date, were eligible for consideration. Only articles published in English were considered eligible. Because the objective was a critical narrative synthesis rather than a systematic review, study selection and evidence appraisal were qualitative, and no formal risk-of-bias instrument or quantitative meta-analysis was applied. Throughout the review, observational associations were distinguished from intervention effects, and evidence concerning cognitive aging or incident dementia was considered separately from outcomes in mild cognitive impairment or established AD.

## 3. Dietary Patterns and Alzheimer’s Disease

Nutritional research has progressively shifted from isolated nutrients toward overall dietary patterns. This approach is particularly relevant to AD because foods are consumed in combination and may exert complementary, synergistic, or opposing effects. Pattern-based analyses also capture nutrient density, degree of processing, energy balance, and the replacement of one food group by another, dimensions that are poorly represented when a single nutrient is examined in isolation [25].

Dietary labels nevertheless conceal clinically important variation. Diets described as Mediterranean, plant-based, vegan, or ketogenic may differ markedly in carbohydrate and fat quality, protein content, micronutrient adequacy, energy density, and reliance on ultra-processed foods. A plant-based pattern centered on legumes, whole grains, vegetables, fruits, nuts, and seeds is metabolically distinct from one dominated by refined grains, sweets, and fried foods. Likewise, a ketogenic intervention emphasizing fish, vegetables, nuts, and unsaturated oils is not equivalent to one based primarily on processed meats, butter, and low-fiber foods. The relevant question is therefore not whether a dietary label is intrinsically protective or harmful, but which foods constitute the diet, what they replace, whether the resulting pattern is nutritionally adequate, and in whom it is applied [20,26,27].

These considerations are particularly important when interpreting observational research, because healthier dietary patterns often cluster with physical activity, higher educational attainment, better healthcare access, and more effective cardiovascular risk management. Most favorable evidence comes from prospective studies of cognitively unimpaired adults or populations at increased risk of cognitive decline. Although informative, these studies remain vulnerable to dietary measurement error, residual confounding, and reverse causality. Early changes in motivation, smell, taste, appetite, and executive function may also influence food selection and meal preparation years before dementia is diagnosed [28,29,30].

Intervention evidence is more limited. Short-term trials can identify changes in metabolism, biomarkers, or selected cognitive outcomes, but they cannot determine whether an intervention prevents dementia or modifies long-term AD progression. The following sections therefore examine the evidence across distinct clinical contexts: cognitive aging and incident dementia, interventions in individuals at increased risk or with mild cognitive impairment, and nutritional strategies evaluated after AD has become clinically established.

Targeted medical nutrition represents a related but distinct approach from whole-diet interventions. Souvenaid^®^ contains Fortasyn Connect, a specific multinutrient formulation providing docosahexaenoic acid (DHA), eicosapentaenoic acid (EPA), uridine monophosphate, choline, phospholipids, selenium, folic acid, and vitamins B12, B6, C, and E [31]. In the 24-month LipiDiDiet randomized trial involving 311 participants with prodromal AD, Fortasyn Connect did not significantly improve the prespecified neuropsychological test battery primary endpoint, although differences favoring the intervention were observed in selected cognitive-functional and MRI secondary outcomes [32]. Longer-term analyses at 36 months reported less decline in cognition, CDR-SB, memory, and brain atrophy measures, although attrition and the smaller number of participants contributing to the extended efficacy analyses warrant cautious interpretation [33]. In contrast, in mild-to-moderate AD, the 24-week S-Connect trial did not demonstrate a significant cognitive benefit [31]. Overall, these findings suggest that specific multinutrient medical nutrition may have greater potential when introduced during prodromal or very early disease, but current evidence does not establish Souvenaid as a disease-modifying treatment or as a substitute for standard AD care [34].

To make the hierarchy of evidence explicit, the following sections distinguish observational associations from human intervention findings and place mechanistic observations in a supporting rather than efficacy-defining role. Where randomized evidence is available, study population, intervention duration, outcomes, and major limitations are emphasized.

## 4. Mediterranean and MIND Dietary Patterns

Among the dietary patterns investigated in relation to cognitive aging, the Mediterranean diet and the Mediterranean–DASH Intervention for Neurodegenerative Delay (MIND) diet have received the greatest epidemiological attention. Both emphasize minimally processed plant foods and unsaturated fats while limiting foods rich in saturated fat, added sugars, and refined carbohydrates [21,35]. However, they arise from different foundations: the Mediterranean diet reflects traditional eating practices in Mediterranean populations, whereas the MIND diet combines selected components of the Mediterranean diet and the Dietary Approaches to Stop Hypertension (DASH) dietary pattern, which was originally developed to prevent and control high blood pressure. The MIND diet further emphasizes foods considered particularly relevant to brain health, including green leafy vegetables, berries, nuts, whole grains, legumes, fish, poultry, and olive oil [21,36].

The Mediterranean diet is characterized by abundant vegetables, fruits, legumes, nuts, and minimally processed cereals; olive oil as the principal culinary fat; regular consumption of fish and seafood; and lower intake of red and processed meat, butter, refined products, and sugar-rich foods [37]. Dairy products, poultry, and eggs are generally consumed in low-to-moderate amounts. Rather than prescribing a rigid macronutrient distribution, the pattern emphasizes the quality and combination of foods consumed over time. The MIND diet retains many of these features but gives particular emphasis to green leafy vegetables, berries, nuts, whole grains, beans, fish, poultry, and olive oil, while limiting red meat, butter, cheese, pastries, sweets, and fried or fast food [21]. Moderate wine consumption was included in the original score, but this historical component should not be interpreted as a recommendation for people who do not drink alcohol to begin doing so.

Adherence to both patterns is generally estimated using food-frequency questionnaires or dietary records. Differences in food categories, intake thresholds, and modified scoring systems reduce comparability across studies. Prospective cohorts have generally associated greater Mediterranean diet adherence with better cognitive performance and lower risks of mild cognitive impairment (MCI), dementia, and AD. A 2024 systematic review and meta-analysis of 21 studies found a modest inverse association with overall dementia and a stronger association with AD, although substantial between-study heterogeneity indicated that the pooled estimates were not uniform across populations [21,38,39].

Observational findings for the MIND diet are also encouraging. Its original prospective evaluation associated greater adherence with lower AD incidence, including among participants with intermediate adherence. A subsequent systematic review found stronger evidence for dementia incidence and cross-sectional global cognition than for longitudinal cognitive decline, domain-specific outcomes, neuroimaging changes, or AD pathology. Much of the favorable evidence also came from North American cohorts, limiting generalizability [40,41]. Clinicopathological observations provide additional biological plausibility. In older adults who underwent postmortem examination, higher Mediterranean and MIND scores were associated with lower global AD pathology and reduced cerebral Aβ burden after adjustment for several demographic and lifestyle factors. These findings remain observational and cannot determine whether diet directly altered amyloid deposition, particularly given self-reported intake, selection of individuals surviving to autopsy, and the possibility that preclinical disease influenced dietary behavior [29,42].

### Human Intervention Evidence

Randomized evidence is substantially more limited than the observational literature. In the PREDIMED cognitive sub-study, 447 cognitively healthy older adults at high cardiovascular risk were randomized to a Mediterranean diet supplemented with extra-virgin olive oil, a Mediterranean diet supplemented with mixed nuts, or advice to follow a lower-fat control diet. Over long-term follow-up, both Mediterranean diet groups showed more favorable changes in selected cognitive outcomes than the control group [43]. However, cognition was evaluated in a selected sub-study of a cardiovascular prevention trial rather than as the primary outcome of the original trial, which limits interpretation specifically for dementia prevention [39,43].

The largest randomized evaluation of the MIND diet enrolled 604 cognitively unimpaired adults aged 65–84 years with a family history of dementia, overweight or obesity, and a suboptimal baseline diet. Participants were assigned to a MIND diet with mild caloric restriction or to a control diet with the same caloric restriction and comparable counseling for three years. Global cognition improved in both groups, with no significant between-group difference, and MRI measures were also similar [44]. The active nature of the control intervention, comparable weight loss, and dietary improvement in both groups may have reduced intervention contrast, but the trial nevertheless did not demonstrate prevention of cognitive decline by the MIND diet.

Shorter controlled studies provide evidence of biological target engagement rather than clinical efficacy. In a four-week randomized feeding trial involving 87 middle-aged adults with normal cognition or MCI, Mediterranean-like and Western-like diets produced different effects on metabolic measures, cerebral perfusion, and CSF AD-related biomarkers, with responses differing according to cognitive status [45]. The short duration, modest sample size, and heterogeneous biomarker responses preclude conclusions regarding dementia prevention or AD progression.

Mechanistically, any potential neurological benefit of Mediterranean- or MIND-type patterns is more plausibly explained by the combined effects of dietary quality on cardiometabolic and vascular health than by a direct action on AD pathology. Controlled dietary evidence supports favorable effects of Mediterranean-type patterns on body weight, glucose regulation, insulin sensitivity, blood pressure, lipid profiles, inflammatory markers, and endothelial function [15]. These changes may reduce vascular and metabolic stress relevant to cognitive vulnerability, including impaired cerebral perfusion and insulin signaling [6,19]. Polyphenols, unsaturated fatty acids, fiber, and other components may additionally influence oxidative and inflammatory pathways, but direct human evidence that these mechanisms translate into reduced cerebral Aβ or tau accumulation remains limited [30]. Mechanistic plausibility should therefore support, rather than substitute for, evidence from clinical outcomes.

These pathways are relevant because they can influence cerebral perfusion, synaptic resilience, glial activation, mitochondrial function, and the handling of Aβ and tau. Nevertheless, direct evidence that either diet slows AD pathology or alters disease progression in humans remains limited [30,41]. Overall, the Mediterranean diet has the broader evidence base, whereas the MIND diet offers a brain-oriented adaptation supported mainly by observational findings. Both provide reasonable models of nutritionally diverse and cardio-metabolically favorable eating, but neither should be interpreted as a treatment for established AD.

## 5. Plant-Based and Vegan Diets

Plant-based diets encompass a spectrum ranging from patterns that prioritize plant foods without excluding animal products to vegetarian diets that omit meat and fish and vegan diets that exclude all animal-derived foods. Evidence derived from broad plant-based indices cannot therefore be extrapolated directly to strict vegan diets. Their nutritional and metabolic effects depend not only on what is excluded but also on the quality of the foods used as replacements [24,46].

### Human Evidence and Intervention Gap

Human evidence relating plant-based dietary patterns to cognition remains predominantly observational. A 2026 systematic review and dose–response meta-analysis including seven prospective studies and 221,380 participants found that greater adherence to overall or healthful plant-based dietary patterns was associated with a lower risk of cognitive impairment and dementia, whereas unhealthful plant-based patterns showed the opposite association. However, substantial heterogeneity and the observational design of the contributing studies limit causal inference [47]. Prospective cohort studies support this quality-dependent interpretation. Among older Chinese adults, greater or increasing adherence to healthful plant-based patterns was associated with lower risk of cognitive impairment, whereas increasing adherence to an unhealthful pattern was associated with less favorable outcomes [48]. A healthful plant-based diet was also associated with slower cognitive decline among African American older adults, although findings were not consistent across all population groups, and the Multiethnic Cohort Study reported modestly lower risks of AD and related dementias with higher overall and healthful plant-based diet scores.

Human intervention evidence is considerably more limited. Trials of plant-based diets have primarily examined body weight, glucose metabolism, cardiovascular risk factors, and systemic inflammation rather than dementia incidence, AD-specific biomarkers, or cognitive decline [48]. No large, long-term randomized trial has demonstrated that adopting a strict vegan diet prevents AD or slows cognitive or functional decline after disease onset. Accordingly, favorable observational findings for high-quality plant-based dietary patterns should not be extrapolated to strict veganism or interpreted as evidence that exclusion of animal-derived foods provides additional protection against AD [48,49]. The biological relevance of high-quality plant-based patterns is likely to be predominantly indirect. Diets rich in minimally processed vegetables, fruits, legumes, whole grains, nuts, seeds, and unsaturated oils may favor body-weight regulation, insulin sensitivity, lipid profiles, vascular function, and systemic inflammatory status, all of which may influence cognitive vulnerability. These potential advantages depend strongly on food quality and should not be attributed simply to the exclusion of animal products.

Nutritional adequacy is central to interpretation because deficiencies could offset the metabolic advantages of a plant-rich diet. Exclusion of animal-derived foods removes the principal natural sources of vitamin B12 and may reduce intake or status of vitamin D, iodine, calcium, zinc, selenium, and the long-chain omega-3 fatty acids eicosapentaenoic acid and docosahexaenoic acid. Vegetarian and vegan adults often consume more fiber, folate, vitamin C, vitamin E, magnesium, and polyunsaturated fats, but may have lower intake or biomarker status for several other nutrients [20,24]. Protein intake is commonly lower than in omnivorous groups but can remain adequate when total energy intake and the variety of protein-rich plant foods are sufficient. Conversely, omnivorous diets are not inherently balanced and may also provide insufficient fiber, folate, vitamin D, calcium, or unsaturated fatty acids [24].

Vitamin B12 requires particular attention because reliable unfortified plant sources are not available. A 2024 systematic review and meta-analysis found lower circulating vitamin B12, higher homocysteine, and greater risk of functional deficiency among vegan adults compared with omnivores. Supplement use was associated with substantially more favorable biomarker profiles [50]. Elevated homocysteine may adversely affect endothelial function, oxidative balance, and cerebrovascular health, while severe or prolonged vitamin B12 deficiency can directly impair neurological function. A strict vegan diet that is not adequately supplemented could therefore increase metabolic and neurological vulnerability rather than reduce it.

Planning becomes especially important in older adults and people with cognitive impairment, who may have reduced appetite, impaired nutrient absorption, medication-related effects, limited food access, difficulty preparing meals, or unintended weight loss. In this context, an unnecessarily restrictive pattern may worsen energy, protein, or micronutrient intake even when its theoretical composition appears favorable. Loss of body weight and muscle mass may further reduce functional reserve and increase frailty, outcomes that are particularly concerning after dementia has become established [23,51].

The available evidence supports increasing minimally processed, nutrient-dense plant foods within a balanced diet. Any potential cognitive benefit is likely to arise from improved insulin sensitivity, lipid metabolism, vascular function, inflammatory regulation, and nutritional status rather than from the exclusion of animal products itself. Current evidence does not establish that strict veganism provides additional protection against AD. Food quality, metabolic effects, and nutritional adequacy are therefore more informative than the vegan label alone.

## 6. Ketogenic Strategies and Alternative Brain Fuels

Unlike Mediterranean, MIND, and plant-based patterns, ketogenic strategies are defined primarily by their ability to increase circulating ketone bodies rather than by a fixed set of foods. Their relevance to AD arises from the early and regionally selective reduction in cerebral glucose metabolism observed along the disease continuum. Brain ketone uptake appears to remain relatively preserved during aging, MCI, and at least some stages of AD, suggesting that ketones may provide an alternative oxidative substrate when glucose utilization is impaired [52,53].

The term “ketogenic intervention” includes metabolically related but nutritionally distinct approaches. A classical ketogenic diet severely restricts carbohydrate and derives most energy from fat, whereas modified ketogenic and modified Atkins diets allow greater protein intake and flexibility. Mediterranean–ketogenic diets combine carbohydrate restriction with fish, vegetables, nuts, and unsaturated oils [52]. Ketone availability may also be increased without a complete ketogenic diet through medium-chain triglycerides (MCTs), caprylic triglyceride formulations, or exogenous ketones. These approaches differ in food composition, degree and duration of ketosis, adherence requirements, and effects on body weight, insulin sensitivity, and circulating lipids. Findings obtained with an MCT formulation should therefore not be generalized to all high-fat or carbohydrate-restricted diets [52].

The metabolic rationale is more direct than for other dietary patterns because ketones can partially bypass impaired cerebral glucose utilization. Ketone bodies enter the brain and are converted into substrates for mitochondrial ATP production, potentially increasing energy availability in neurons and glial cells when glucose metabolism is insufficient. This energetic compensation could support membrane function, neurotransmission, and synaptic activity without necessarily correcting the underlying causes of cerebral hypometabolism. Ketogenic interventions should therefore be understood primarily as a strategy to supplement brain energy metabolism rather than as evidence that AD pathology has been reversed [54,55].

### Human Intervention Evidence

Clinical studies demonstrate reliable metabolic target engagement, but cognitive and functional findings remain heterogeneous. In a randomized six-month trial, 52 individuals with MCI received 30 g/day of ketogenic MCT or placebo. Brain ketone metabolism increased markedly with the intervention while glucose uptake remained unchanged, and improvements were reported in selected measures of episodic memory, language, executive function, and processing speed [54]. The trial was small, cognition was not the primary metabolic endpoint, and approximately one quarter of participants did not complete the intervention.

Short dietary trials have provided additional signals. In 23 older adults with MCI, a six-week very-low-carbohydrate diet improved verbal memory compared with a high-carbohydrate control diet, and memory performance correlated with circulating ketone concentrations [56]. In established AD, however, larger and longer studies have been less convincing. A 26-week randomized trial of AC-1204 in 413 participants with mild-to-moderate AD found no significant improvement in ADAS-Cog, global clinical status, or functional outcomes among the prespecified APOE ε4 non-carrier population [57].

Dietary ketogenic trials have likewise produced mixed clinical findings. In a randomized crossover trial involving 26 people with AD, a 12-week modified ketogenic diet improved activities of daily living and quality of life relative to the comparison diet, but cognition did not differ significantly [58]. A more recent 12-week feasibility trial randomized 38 individuals with MCI attributed to AD to a modified Atkins or control diet; attrition was substantial, only two participants met the prespecified ketosis-based adherence criterion, and no significant memory benefit was demonstrated [59]. Collectively, these studies indicate that ketosis can be achieved and may alter cerebral energy availability, but they do not establish durable cognitive benefit or modification of AD progression.

Ketogenic interventions may also influence AD-related vulnerability indirectly through changes in systemic metabolism. Carbohydrate restriction commonly reduces postprandial glucose and insulin concentrations and may improve peripheral insulin sensitivity, particularly when accompanied by weight loss. Reduced hyperinsulinemia could lessen metabolic and vascular stress and potentially support brain insulin signaling, which participates in neuronal survival and synaptic plasticity [19,60]. However, current trials cannot determine whether reported cognitive or biomarker changes result from ketosis itself, lower glucose exposure, improved insulin sensitivity, caloric restriction, weight loss, or the removal of refined carbohydrates.

Ketone bodies may additionally act as signaling metabolites rather than only as energy substrates. Experimental evidence suggests that they can influence mitochondrial redox balance, oxidative stress responses, inflammatory signaling, and cellular pathways involved in metabolic adaptation. These effects could theoretically reduce mitochondrial dysfunction and chronic activation of inflammatory pathways that contribute to neuronal and synaptic injury. Nevertheless, it remains uncertain whether the concentrations and durations of ketosis achieved in clinical studies are sufficient to produce meaningful changes in human neuroinflammation or AD pathology [52,61,62].

Effects on lipid metabolism are particularly dependent on the composition of the diet. A ketogenic pattern based on fish, nuts, vegetables, olive oil, and other unsaturated fats may have a different vascular and inflammatory profile from one dominated by butter, processed meats, and foods low in fiber. In some individuals, high intakes of saturated fat may increase low-density lipoprotein cholesterol and potentially worsen long-term vascular risk. Because vascular injury can coexist with and amplify the clinical expression of AD pathology, unfavorable lipid changes could offset any short-term energetic benefit of ketosis. Dietary composition, rather than ketosis alone, is therefore critical when evaluating the neurological implications of these interventions [6,26,63].

Changes in the gut microbiota, short-chain fatty acid production, and inflammatory metabolites may provide another indirect pathway, but the direction and clinical relevance of these changes remain uncertain. Severe carbohydrate restriction can reduce the intake of fiber-rich foods, whereas Mediterranean–ketogenic approaches may better preserve vegetables, nuts, and unsaturated fats. This variability further limits generalization across ketogenic studies [64].

Safety and feasibility are especially important in older adults. Gastrointestinal discomfort, diarrhea, constipation, and reduced appetite are common with MCT formulations. Restrictive ketogenic diets may contribute to unintended weight loss, inadequate fiber or micronutrient intake, and unfavorable lipid changes, depending on the foods and fats selected. These concerns are amplified by frailty, sarcopenia, dysphagia, diabetes, renal or hepatic disease, and pre-existing weight loss. In people with established dementia, any potential metabolic benefit must therefore be balanced against the risks of reduced intake, loss of lean mass, treatment burden, and poor adherence [23,52].

Nutritional ketosis should be distinguished from diabetic ketoacidosis, but clinical monitoring remains necessary when metabolic comorbidities or glucose-lowering medications increase risk. Overall, ketogenic strategies offer a biologically plausible means of partially compensating for impaired cerebral glucose utilization. Their effects may also involve changes in insulin signaling, lipid metabolism, oxidative stress, inflammation, and mitochondrial function. However, the relative contribution of each pathway remains unresolved, and improvements in metabolic biomarkers should not be interpreted as evidence of disease modification. The principal human intervention studies evaluating dietary patterns, specific multinutrient medical nutrition, and ketogenic strategies are summarized in Table 1 [52,65,66].

## 7. Vitamin B12, Folate, and One-Carbon Metabolism

Vitamin B12 and folate are central to one-carbon metabolism, which supports nucleotide synthesis, amino acid homeostasis, methylation, and cellular redox balance. Disturbance of this network may compromise neural and vascular function, providing a plausible link between micronutrient status and cognitive vulnerability. Vitamin B12 is a cofactor for methionine synthase, which transfers a methyl group from 5-methyltetrahydrofolate to homocysteine, regenerating methionine and tetrahydrofolate. Methionine is subsequently converted into S-adenosylmethionine, the principal methyl donor for reactions involving DNA, RNA, proteins, phospholipids, and neurotransmitter metabolism. Vitamin B12 is also required by methylmalonyl-CoA mutase. Inadequate availability therefore leads to accumulation of homocysteine and methylmalonic acid and may interfere with methylation, myelin maintenance, membrane metabolism, and cellular energy homeostasis [50,67,68,69,70].

Folate supplies one-carbon units required for nucleotide synthesis and homocysteine remethylation, while vitamin B6 participates in the transsulfuration pathway that converts homocysteine to cysteine and supports glutathione synthesis. These nutrients should not be considered independently [71]. Low vitamin B12 or folate status, insufficient vitamin B6, impaired renal function, genetic variation, and several clinical conditions may all increase plasma total homocysteine. Conversely, homocysteine is nonspecific and cannot diagnose vitamin B12 deficiency on its own [72].

Natural vitamin B12 sources are largely restricted to animal-derived foods, although fortified foods and supplements provide effective alternatives. For people avoiding all animal-derived foods, fortification or supplementation is essential. In older adults, deficiency more often reflects impaired release or absorption of food-bound cobalamin than complete dietary exclusion. Atrophic gastritis, pernicious anemia, gastric or intestinal surgery, inflammatory bowel disease, and long-term metformin use can reduce vitamin B12 availability [73]. Assessment is not always straightforward. Total serum vitamin B12 concentrations below approximately 148 pmol/L are commonly considered consistent with deficiency, but thresholds vary, and neurological manifestations may occur at borderline concentrations. Holotranscobalamin reflects the fraction available for cellular uptake, whereas methylmalonic acid and homocysteine provide evidence of functional impairment. Interpretation should account for renal function, folate and vitamin B6 status, age, thyroid function, smoking, alcohol use, and medication exposure [72,74].

Folate supplementation may improve hematological abnormalities while neurological injury from vitamin B12 deficiency continues, making concurrent assessment important in at-risk individuals. Vitamin B12 deficiency can cause peripheral neuropathy, impaired proprioception, gait disturbance, subacute combined degeneration of the spinal cord, cognitive changes, and neuropsychiatric symptoms. Some manifestations become only partially reversible when treatment is delayed. Cognitive impairment caused or aggravated by severe vitamin B12 deficiency should not be considered equivalent to AD. Vitamin B12 assessment is clinically relevant because deficiency is a potentially reversible or contributory condition, not because a low concentration establishes the cause of a neurodegenerative syndrome [68,75,76].

The direct relationship between circulating vitamin B12 and AD has been inconsistent. Some observational studies have reported lower vitamin B12 or folate concentrations in AD, whereas others found no independent association after adjustment for age, renal function, nutritional status, and vascular risk [77,78]. Evidence relating elevated homocysteine to cognitive outcomes is more consistent. A dose–response meta-analysis found an approximately 15% higher relative risk of Alzheimer-type dementia for each 5 μmol/L increase in blood homocysteine, while a later meta-analysis also reported increased AD risk but identified heterogeneity and possible publication bias. Homocysteine may therefore identify vascular or neurological vulnerability without necessarily acting as an independent causal driver [79,80,81].

Elevated homocysteine provides biological plausibility for these findings because it is associated with endothelial dysfunction, oxidative stress, cerebrovascular injury, and disturbances in one-carbon metabolism [82]. Preclinical studies additionally link B-vitamin deficiency or hyperhomocysteinemia to amyloid processing, tau phosphorylation, and synaptic dysfunction, but these observations should not be interpreted as evidence that vitamin deficiency initiates AD in humans.

### Human Intervention Evidence

The strongest intervention signal comes from VITACOG, a single-center randomized double-blind trial involving 271 adults aged over 70 years with MCI. Participants received folic acid (0.8 mg/day), vitamin B12 (0.5 mg/day), and vitamin B6 (20 mg/day) or placebo for 24 months. Among the 187 participants who underwent serial MRI, the annual rate of whole-brain atrophy was lower with B-vitamin treatment, with the largest effect among participants with elevated baseline homocysteine [83,84]. Secondary analyses of the same trial reported benefits in selected cognitive and clinical outcomes, again particularly among participants with higher baseline homocysteine [84]. These findings suggest that baseline metabolic status may modify treatment response, but VITACOG was conducted at a single center, imaging was available only in a subset, and MCI was not characterized using contemporary amyloid or tau biomarkers. The trial therefore did not establish prevention of biologically defined AD or progression from MCI to AD dementia.

Potential interactions with long-chain omega-3 fatty acids may also modify the response to B-vitamin interventions. In a post hoc analysis of the OmegAD randomized trial, the cognitive response to omega-3 supplementation varied according to baseline homocysteine, with more favorable effects among participants with lower homocysteine concentrations, suggesting that B-vitamin status may influence omega-3 responsiveness [85]. Conversely, a post hoc analysis of the FACIT trial found that the cognitive effect of folic acid supplementation also depended on baseline omega-3 fatty acid status [86]. Together with the broader literature on omega-3 supplementation, these observations support the possibility of nutrient–nutrient interactions, but they are based largely on secondary analyses and do not establish that combined omega-3 and B-vitamin supplementation prevents or treats AD [85,86,87].

Evidence in clinically diagnosed AD is less convincing. A 2024 meta-analysis of five randomized trials found that combined vitamin B12 and folic acid lowered homocysteine and produced a small improvement in Mini-Mental State Examination scores after six months, but no significant benefit in ADAS-Cog or activities of daily living. Small samples, heterogeneous doses, short follow-up, and discordant cognitive and functional outcomes limit interpretation. Vitamin B12 replacement is indicated when biochemical or clinical deficiency is present, particularly because delayed treatment may permit irreversible neurological injury. Routine high-dose B-vitamin supplementation cannot currently be recommended as a strategy for preventing or treating AD in nutritionally sufficient individuals. Any neurological benefit is more likely to occur when an identifiable metabolic limitation, such as deficiency or elevated homocysteine, is present.

## 8. Vitamin D and Alzheimer’s Disease

Vitamin D raises a related but distinct question: whether correction of low status can influence cognition or AD-related outcomes. Low vitamin D status is common in older adults and has repeatedly been associated with cognitive impairment and dementia. However, vitamin D concentrations are strongly influenced by mobility, sunlight exposure, adiposity, chronic disease, and general health, making their relationship with AD potentially bidirectional [88,89].

Vitamin D is obtained through cutaneous synthesis and, to a lesser extent, from food and supplements. Vitamin D2 and vitamin D3 are hydroxylated in the liver to 25-hydroxyvitamin D [25(OH)D], the principal circulating marker of status, and subsequently converted—primarily in the kidney but also locally in other tissues—to 1,25-dihydroxyvitamin D [1,25(OH)2D] [90]. The active metabolite signals mainly through the vitamin D receptor, which is expressed in neurons, astrocytes, microglia, and vascular cells in brain regions relevant to cognition. Receptor expression and mechanistic plausibility do not, however, demonstrate that supplementation improves cognitive outcomes. Older adults are susceptible to low 25(OH)D because cutaneous synthesis declines with age and may be further limited by reduced outdoor activity, institutionalization, low dietary intake, malabsorption, obesity, liver or kidney dysfunction, and medication use. Many of these factors are also common in cognitive impairment and dementia. Serum 25(OH)D is preferred for assessing vitamin D status because it reflects both cutaneous production and intake and has a longer half-life than 1,25(OH)2D [91,92,93,94]. Interpretation remains complicated by assay variation, season, adiposity, vitamin D-binding protein, and the clinical outcome used to define adequacy.

Earlier guidelines often defined deficiency as 25(OH)D below 20 ng/mL (50 nmol/L) and insufficiency as 20–30 ng/mL. These thresholds were developed mainly for skeletal outcomes and should not be assumed to represent cognitive targets. The Endocrine Society’s 2024 guideline no longer endorses universal sufficiency, insufficiency, or deficiency thresholds for disease prevention in otherwise healthy populations because randomized trials have not established outcome-specific target concentrations [95,96]. Experimental studies suggest several pathways through which vitamin D could influence the aging brain. Vitamin D receptor signaling may modulate immune responses, microglial activation, cytokine production, calcium homeostasis, mitochondrial function, antioxidant defenses, neurotrophic signaling, and vascular integrity. Active vitamin D has enhanced macrophage-mediated Aβ phagocytosis in cells obtained from people with AD, and preclinical models have reported changes in Aβ production, degradation, or clearance after modulation of vitamin D pathways [97,98]. These findings support biological plausibility but do not demonstrate that oral supplementation removes cerebral amyloid or improves clinical outcomes. Vitamin D receptor signaling may itself become dysregulated in AD, suggesting that biological response could depend on cellular context and disease stage.

Prospective studies have repeatedly associated low 25(OH)D with greater risk of cognitive impairment, dementia, and AD. In 1658 initially dementia-free older adults, moderate and severe deficiency predicted greater subsequent risk of all-cause dementia and AD. A 2024 meta-analysis of 23 prospective studies reported approximately 42% greater risk of dementia, 57% greater risk of AD, and 34% greater risk of cognitive impairment among participants classified as vitamin D deficient, with a nonlinear relationship driven mainly by the lowest concentrations [88]. These associations are difficult to interpret causally. Low vitamin D clusters with physical inactivity, obesity, diabetes, cardiovascular disease, frailty, social isolation, poor diet, and limited outdoor exposure. Subtle prodromal changes in motivation, executive function, mobility, and social activity may also reduce sunlight exposure and food intake before dementia is diagnosed.

Mendelian randomization studies have produced mixed findings, with some support for an effect of marked genetically predicted deficiency but no consistent evidence that progressively higher genetically predicted 25(OH)D improves cognition or lowers AD risk in the general population [99,100].

### Human Intervention Evidence

Randomized trials have not reproduced the magnitude of the associations observed in cohort studies. In two cognitive ancillary studies of the VITAL trial, vitamin D3 at 2000 IU/day did not significantly slow decline in global cognition over approximately two to three years; the analyses included 3424 participants assessed by telephone and 794 assessed in person [101]. Similarly, the Finnish Vitamin D Trial included 2492 dementia-free older adults randomized to placebo, 1600 IU/day, or 3200 IU/day of vitamin D3 for up to five years and found no significant difference in incident dementia or AD, although the number of dementia events was small and baseline vitamin D status was generally sufficient [102].

VitaMIND specifically addressed a population with lower vitamin D status. In this 24-month randomized trial, 620 adults aged ≥50 years with mild-to-moderate vitamin D deficiency and early cognitive impairment received vitamin D3 or placebo. Supplementation did not improve the primary executive-function outcome or secondary measures of cognition, function, or well-being [103]. Evidence in clinically established AD is more limited. A 12-month randomized trial in 210 participants with AD reported improvements in selected cognitive measures and peripheral Aβ-related biomarkers with 800 IU/day of vitamin D, but these findings have not been consistently reproduced, and peripheral biomarker changes cannot establish modification of cerebral AD pathology [104]. Overall, randomized evidence does not currently support vitamin D supplementation as a strategy to prevent or modify AD, although documented deficiency should still be treated for established clinical indications.

Vitamin D is generally well tolerated at conventional replacement doses, but unnecessary high-dose supplementation can cause hypercalcemia, hypercalciuria, nephrolithiasis, renal impairment, and medication interactions. When supplementation is clinically indicated in older adults, current guidance generally favors daily lower-dose administration over large intermittent doses. Documented deficiency should be evaluated and treated because vitamin D is important for skeletal health, muscle function, and mobility. Key human intervention studies evaluating B-vitamin and vitamin D supplementation in relation to cognitive impairment and AD are summarized in Table 2.

Overall, the nutritional approaches examined in this review differ substantially in their evidentiary support and proposed mechanisms. Mediterranean and MIND dietary patterns have the strongest observational associations with healthier cognitive aging, although randomized trials have not consistently demonstrated prevention of cognitive decline. Healthful plant-based patterns may provide benefits through favorable effects on insulin sensitivity, lipid metabolism, vascular function, inflammation, and oxidative stress, but strict vegan diets have not shown additional protection and require careful nutritional planning. Ketogenic strategies may partially compensate for impaired cerebral glucose utilization by increasing ketone availability, yet their cognitive effects remain inconsistent, and their long-term safety and efficacy are uncertain. Similarly, adequate vitamin B12, folate, and vitamin D status is important for neurological and general health, but supplementation appears most justified for correcting deficiency rather than as a disease-modifying treatment for AD. A comparative summary of the evidence, proposed mechanisms, limitations, and clinical implications of these dietary and micronutrient strategies is presented in Table 3.

## 9. Cross-Cutting Modifiers of Nutritional Response

The clinical relevance of nutritional interventions is likely to depend strongly on disease stage and baseline nutritional and metabolic status. Strategies aimed at reducing long-term cardiometabolic risk in cognitively healthy individuals are fundamentally different from interventions initiated after MCI or AD has developed, while correction of an established nutrient deficiency is biologically distinct from supplementation in nutritionally sufficient individuals [30,106]. These distinctions may explain part of the heterogeneity observed across nutritional studies and should guide both trial design and interpretation.

Biological factors may further modify treatment response. APOE ε4 is a plausible modifier of responses to dietary fat, omega-3 fatty acids, Mediterranean dietary patterns, and ketogenic interventions, but current evidence is inconsistent and insufficient to support genotype-specific dietary prescriptions [107]. Age, biological sex, body composition, insulin resistance, vascular comorbidity, renal function, medication use, and baseline nutrient status may also influence nutritional responses [106,108]. These factors should increasingly be incorporated prospectively into nutritional trials rather than explored only through underpowered post hoc subgroup analyses.

Practical feasibility is another major determinant of real-world effectiveness. Palatability, cost, culture, food availability, gastrointestinal tolerance, cognitive capacity, functional dependence, and caregiver support determine whether a nutritional intervention can be sustained. These considerations become increasingly important as dementia progresses, when prevention of weight loss, malnutrition, dehydration, sarcopenia, and feeding difficulties may become more clinically relevant than adherence to a theoretically neuroprotective dietary pattern [23]. Nutritional care should therefore be individualized according to disease stage, nutritional status, metabolic phenotype, functional capacity, and goals of care.

## 10. Clinical Implications

The clinical implications outlined below represent an evidence-informed synthesis of randomized trials, observational studies, and established nutritional guidance rather than recommendations derived from a single intervention study. When direct randomized evidence is available, its findings are stated explicitly; where trial evidence is absent or inconsistent, practical implications are presented as cautious clinical interpretation. Accordingly, these statements should not be interpreted as evidence that any dietary pattern or nutritional intervention independently prevents or modifies AD [23,106]. The available evidence supports an important but clearly delimited role for nutrition across the AD continuum. Dietary care may contribute to long-term cardiovascular and metabolic risk reduction, correct clinically relevant deficiencies, and preserve nutritional status after cognitive impairment develops. It should complement rather than replace established diagnostic, pharmacological, rehabilitative, and supportive care [30].

Based on the overall synthesis of the available evidence, a clinically reasonable approach for cognitively healthy adults and people at increased risk of dementia is to improve overall dietary quality while addressing cardiometabolic conditions. Mediterranean- and MIND-type patterns can serve as practical models for this purpose because they emphasize minimally processed plant foods, unsaturated fats, whole grains, legumes, nuts, and fish. However, this practical interpretation should not be taken as evidence that adherence to either named dietary pattern independently prevents AD. Observational findings are generally favorable, but randomized evidence remains limited, and the largest randomized MIND diet trial found no significant between-group difference in cognitive decline over three years [15,21].

Importantly, any potential effect on long-term cognitive resilience is likely to depend on consistent dietary exposure maintained over years rather than on short-term adoption of a healthier diet. Because vascular, metabolic, and neurodegenerative processes develop gradually across the life course, brief dietary interventions may be insufficient to produce measurable changes in dementia incidence or AD progression. Earlier initiation and sustained adherence may therefore be more relevant than temporary dietary modification, although the duration and timing required to influence cognitive outcomes remain uncertain [12,30,106]. Nutrition should be integrated with physical activity, smoking cessation, adequate sleep, social participation, and appropriate management of hypertension, diabetes, and dyslipidemia.

Current evidence does not justify recommending strict veganism or ketogenic therapy specifically for AD prevention. A vegan diet can be nutritionally adequate but requires a reliable vitamin B12 source and attention to protein, vitamin D, iodine, calcium, zinc, and long-chain omega-3 fatty acids. Ketogenic interventions remain experimental and require consideration of baseline weight, lipid profile, diabetes treatment, kidney and liver function, gastrointestinal tolerance, food preferences, and the ability to sustain carbohydrate restriction [23,48]. Once MCI or dementia is present, nutritional care should begin with assessment rather than prescription. The ESPEN guideline recommends routine screening for malnutrition and low-intake dehydration, followed by comprehensive evaluation and monitoring when risk is identified. Relevant information includes recent weight change, food and fluid intake, appetite, dietary restrictions, gastrointestinal symptoms, oral health, chewing and swallowing, mobility, functional independence, medications, comorbidities, and caregiver support [23,109]. Serial changes in weight, meal completion, hydration, muscle strength, and function are often more informative than a single body mass index measurement. Potentially reversible causes of poor intake should be sought before introducing further dietary restriction. These may include pain, depression, infection, constipation, medication effects, dry mouth, dental problems, sensory impairment, difficulty recognizing or preparing food, environmental distraction, and social isolation. Laboratory testing should be guided by symptoms and risk factors. Vitamin B12 assessment is particularly relevant in vegan or highly restrictive diets, anemia, neurological manifestations, gastrointestinal disease or surgery, pernicious anemia, and prolonged metformin use. Borderline or discordant results may require methylmalonic acid or homocysteine, interpreted in the context of renal function and folate status [23]. Vitamin D testing is similarly most appropriate when there is a recognized clinical indication, such as malabsorption, osteoporosis, disorders of calcium metabolism, marked restriction of sunlight exposure, or concern about deficiency. Supplementation should correct an identified clinical problem rather than be prescribed with the expectation of reversing cognitive decline.

Nutritional priorities change as cognitive and functional impairment progresses. In early disease, the emphasis may remain on dietary variety, cardiometabolic health, and prevention of nutritional decline. In moderate or advanced dementia, preserving body weight, muscle mass, hydration, comfort, and the social meaning of eating often becomes more important than strict adherence to a theoretically neuroprotective pattern. Rapid intentional weight loss, caloric restriction, or highly restrictive diets should generally be avoided in people with frailty, sarcopenia, recurrent illness, or unintended weight loss unless there is a compelling indication and close professional supervision. Adequate protein and energy intake deserve particular attention because loss of muscle aggravates weakness, falls, dependence, and reduced participation in rehabilitation. Mealtime support is a clinical intervention. Familiar routines, sufficient time, appropriate lighting, limited distraction, adaptive utensils, oral hygiene, and assistance that preserves independence can improve intake. Caregivers should be included because they determine much of the practical feasibility of meal planning and supplementation. Suspected dysphagia requires timely assessment and individualized strategies for positioning, pacing, texture modification, and fluid consistency. Modified textures should remain nutritionally adequate and acceptable. Oral nutritional supplements may be useful when regular food and assistance do not meet nutritional requirements, but their purpose is to improve or maintain nutritional status rather than cognition [23].

Artificial nutrition and hydration require individualized clinical and ethical judgment. Temporary enteral nutrition may be considered in mild or moderate dementia when insufficient intake is caused mainly by a potentially reversible condition and oral strategies cannot meet requirements. Enteral feeding should not be initiated routinely in severe dementia, and artificial nutrition or hydration is generally not indicated in the terminal phase. Decisions should consider prognosis, expected benefit, complications, previously expressed preferences, caregiver perspectives, and goals of care. Nutritional care is best delivered through a multidisciplinary process involving physicians, dietitians, nurses, speech and language therapists, occupational therapists, dentists, and caregivers when appropriate. The aim is not to impose a universal diet for AD, but to match nutritional care to disease stage, metabolic health, nutritional status, function, preferences, and realistic goals. Stage-specific nutritional objectives and clinical priorities are summarized in Table 4.

## 11. Current Limitations and Future Directions

The main limitation of the current literature is not simply insufficient evidence, but a recurrent mismatch between the intervention studied, the biological process targeted, the population enrolled, and the outcome used to judge efficacy. Dietary patterns are frequently reduced to broad labels that conceal substantial variation in food quality, nutrient adequacy, processing, energy intake, and metabolic exposure. Consequently, studies nominally evaluating the same diet may test meaningfully different interventions. Future research should therefore define dietary exposure quantitatively and verify that the intended nutritional or metabolic target was achieved rather than relying primarily on adherence scores or self-reported dietary labels. Because this was a targeted critical narrative review rather than an exhaustive systematic review of all nutritional exposures, potentially relevant dietary components outside the selected domains may be underrepresented, and this scope should be considered when interpreting the synthesis.

Causal interpretation is further complicated by measurement error, residual confounding, and reverse causality. Diet is embedded within broader socioeconomic, behavioral, and clinical contexts, while preclinical neurodegeneration may itself alter appetite, sensory perception, body weight, food choice, and the ability to prepare meals. More informative longitudinal designs will require repeated dietary and cognitive assessments, objective nutritional biomarkers, longer follow-up, and analytical strategies that distinguish antecedent exposure from dietary changes occurring during the prodromal phase of disease.

Biological characterization of participants is equally important. Clinical categories such as MCI and dementia encompass heterogeneous and often mixed pathologies, limiting the ability to identify nutrition-specific effects on AD. The incorporation of amyloid, tau, and neurodegeneration biomarkers, supported increasingly by scalable plasma markers such as phosphorylated tau 217, may reduce this heterogeneity and allow nutritional interventions to be evaluated in biologically defined populations. However, biomarker responses should not be interpreted as evidence of clinical benefit unless accompanied by meaningful cognitive or functional outcomes.

Disease stage should determine both the intervention and the expected outcome. Preventing cognitive decline in healthy adults, delaying symptoms in biomarker-positive individuals, modifying established disease, and supporting nutrition in advanced dementia are fundamentally different objectives. Short-term changes in metabolism or cognition cannot establish dementia prevention, just as improvements in nutritional status should not be presented as disease modification. Trials must therefore align their duration and endpoints with the time course and mechanism they propose to influence.

Heterogeneity in baseline nutritional and metabolic status may also explain inconsistent findings. Nutrient supplementation is unlikely to produce uniform effects in deficient and sufficient individuals, while metabolic interventions may be more relevant in participants with obesity, insulin resistance, or other targetable abnormalities. Precision nutrition offers a useful conceptual framework, but subgroup analyses involving genotype, sex, age, body composition, medications, microbiota, or comorbidity should be prespecified, adequately powered, and independently replicated before being translated into personalized recommendations.

Future trials also need stronger evidence that participants received and sustained the intended intervention. Adherence should be evaluated through objective biomarkers or metabolic measures whenever possible, and interpreted alongside acceptability, adverse effects, cost, cultural compatibility, and caregiver burden. Long-term feasibility is especially important because an intervention that is metabolically effective but difficult to sustain is unlikely to alter outcomes that develop over decades.

Nutrition should increasingly be studied within multidomain prevention, while avoiding attribution of the combined effect solely to diet. Recent multidomain trials suggest that modest cognitive benefits may emerge when dietary improvement is integrated with physical activity, vascular-risk control, cognitive stimulation, sleep, and social engagement. Future studies should retain sufficient measurement of each component to determine whether diet provides an independent, synergistic, or primarily supportive contribution.

Greater population diversity and explicit safety assessment are also essential. Findings derived mainly from well-resourced populations may not generalize across differences in food access, cultural dietary practices, cardiometabolic risk, ancestry, healthcare availability, or baseline nutritional status. Potential harms—including unintended weight loss, nutrient inadequacy, metabolic deterioration, medication interactions, gastrointestinal intolerance, and increased caregiver workload—should be considered part of efficacy rather than treated as secondary concerns.

The most realistic goal for the field is therefore not to identify a universal diet for AD. Progress will depend on determining which nutritional abnormalities are modifiable, which biological or clinical subgroups are most likely to benefit, at what stage intervention remains meaningful, and whether observed effects translate into sustained improvements in cognition, function, nutritional status, or quality of life.

## 12. Discussion

The available evidence supports a clinically relevant but circumscribed role for nutrition across the AD continuum. Apparent inconsistencies in the literature partly reflect the evaluation of heterogeneous dietary exposures in populations that differ in nutritional status, metabolic phenotype, pathological substrate, and disease stage. Accordingly, nutrition is more appropriately interpreted as a potential modifier of physiological resilience and comorbid risk than as a stand-alone treatment for AD pathology. This distinction is increasingly emphasized in contemporary frameworks for nutrition and dementia prevention, which highlight the importance of disease stage, baseline nutritional status, and individual metabolic characteristics when interpreting dietary effects [30,106].

Across dietary patterns, food quality and nutritional adequacy appear more informative than the dietary label itself. Mediterranean-type patterns currently have the most consistent evidence for favorable cardiometabolic effects and have been associated with healthier cognitive aging, but randomized evidence does not establish specific prevention of AD. Importantly, the largest randomized trial of the MIND diet found no significant between-group benefit in cognitive decline despite favorable observational findings [44]. These observations reinforce the need to distinguish epidemiological associations from intervention effects and to avoid interpreting a nutritionally favorable dietary pattern as a disease-specific therapy.

This distinction is particularly relevant to ketogenic interventions and micronutrient supplementation. Ketogenic strategies can increase cerebral ketone availability and may partially compensate for impaired glucose utilization, but available clinical trials remain relatively small and heterogeneous, and durable effects on cognition, function, or AD progression have not been established [52]. Similarly, correction of vitamin B12, folate, or vitamin D inadequacy can restore important physiological functions and may be clinically necessary, but correction of deficiency is conceptually different from modification of the neurodegenerative process. For B-vitamin interventions in particular, the available evidence suggests that baseline metabolic status, including homocysteine concentrations, may influence treatment response [30,82].

Timing is likely to be a major determinant of nutritional effects. Dietary exposures acting over many years may influence later cognitive vulnerability indirectly through vascular health, insulin sensitivity, adiposity, and other cardiometabolic pathways. Vascular dysfunction and impaired insulin signaling can coexist with and potentially amplify the clinical expression of AD pathology, providing a rationale for addressing these factors even when doing so does not directly alter amyloid or tau accumulation [6,19]. Once substantial neurodegeneration is established, however, interventions aimed primarily at improving metabolic health may be less likely to produce measurable disease modification. This stage-dependent interpretation is consistent with broader dementia-prevention frameworks emphasizing cumulative and multidomain risk reduction across the life course [12,30].

After dementia develops, the clinical priorities of nutritional care progressively shift. Preservation of body weight, muscle mass, hydration, safe oral intake, functional capacity, and quality of life may become more relevant than adherence to a theoretically neuroprotective dietary pattern. Current ESPEN recommendations similarly emphasize prevention and treatment of malnutrition and dehydration, individualized nutritional assessment, mealtime support, and adaptation of nutritional care to disease severity and goals of care [23]. These supportive outcomes should not be regarded as secondary simply because they do not modify the core neuropathology of AD.

Taken together, the evidence supports separating four concepts that are frequently conflated in the nutrition–AD literature: epidemiological association, correction of nutritional deficiency, metabolic compensation, and modification of AD pathology. Each requires different study designs, populations, intervention durations, biomarkers, and clinical endpoints. Future progress will therefore depend less on identifying a universal “AD diet” and more on determining which nutritional or metabolic abnormalities are modifiable, which individuals are most likely to benefit, and at what stage intervention remains clinically meaningful [30,106]. Nutrition should ultimately be integrated into multidomain prevention and person-centered dementia care, with claims proportional to the type and strength of the available evidence [12,23,30].

## 13. Conclusions

Nutrition has a clinically relevant but circumscribed role across the AD continuum. The evidence reviewed supports five principal conclusions:

1. Nutrition is best understood as a modifier of physiological resilience and comorbid risk rather than as a direct treatment for AD pathology.
2. No dietary pattern, restrictive regimen, or isolated supplement has demonstrated independent prevention or modification of AD progression.
3. The potential value of a diet depends more on its nutritional quality, metabolic suitability, sustainability, and practical feasibility than on the label used to describe it.
4. Nutrient deficiencies should be identified and corrected because of their broader neurological and functional consequences, without assuming that supplementation beyond adequacy will improve cognition.
5. Nutritional goals must change with disease stage: long-term cardiometabolic risk reduction is most relevant before substantial neurodegeneration, whereas preservation of intake, hydration, muscle function, comfort, and quality of life becomes increasingly important as dementia progresses.

The central implication is that nutritional interventions should be matched to the individual’s biological vulnerabilities, nutritional status, functional capacity, disease stage, and goals of care. Nutrition should therefore be integrated into multidomain prevention and person-centered dementia care, rather than presented as a stand-alone disease-modifying strategy.

## Figures and Tables

**Table 1:** Key Human Intervention Studies of Dietary Patterns, Specific Medical Nutrition, and Ketogenic Strategies Relevant to Alzheimer’s Disease.

Study	Design, Population and Disease Stage	Intervention vs. Comparator	Duration	Main Outcomes	Main Findings	Relevant Limitations
Valls-Pedret et al., 2015 [43].	Randomized cognitive substudy of PREDIMED; *n* = 447 randomized (334 with end-of-study cognitive assessment); cognitively healthy older adults at high cardiovascular risk.	Mediterranean diet + extra-virgin olive oil or Mediterranean diet + mixed nuts vs. lower-fat dietary advice	≈4 years	Global cognition and memory/executive composites	Both Mediterranean diet groups showed more favorable changes in selected cognitive outcomes than the control group	Cognition was assessed in a selected substudy and was not the primary outcome of the original cardiovascular trial
Barnes et al., 2023 [44].	Randomized controlled trial; *n* = 604; cognitively unimpaired adults aged 65–84 years with a family history of dementia, overweight/obesity, and a suboptimal baseline diet.	MIND diet + mild caloric restriction vs. control diet with equivalent caloric restriction and counseling	3 years	Global cognition, cognitive domains, MRI measures	Cognition improved in both groups, with no significant between-group difference; MRI outcomes were also similar	Active control, similar weight loss, and dietary improvement in both groups may have reduced intervention contrast
Hoscheidt et al., 2022 [45].	Randomized controlled feeding trial; *n* = 87 (56 with normal cognition and 31 with MCI); middle-aged adults.	Mediterranean-like diet vs. Western-like diet	4 weeks	CSF AD biomarkers, cerebral perfusion, metabolic measures, cognition	Diet-related changes in metabolic, perfusion, and biomarker outcomes differed according to cognitive status	Small sample, very short duration, and biomarker changes cannot establish clinical efficacy or dementia prevention
Shah et al., 2013 [31].	Randomized controlled trial; *n* = 527; participants with mild-to-moderate AD receiving standard AD medications.	Souvenaid/Fortasyn Connect vs. isocaloric control	24 weeks	Cognition and functional outcomes	No significant cognitive benefit was demonstrated	Relatively short treatment period
Soininen et al., 2017; 2021 [32,33].	Randomized double-blind controlled trial; *n* = 311; participants with prodromal AD.	Fortasyn Connect/Souvenaid vs. isocaloric control	24 months; extension to 36 months	Neuropsychological test battery, CDR-SB, memory, MRI brain atrophy measures	Primary cognitive endpoint was neutral at 24 months; selected secondary outcomes and several 36-month cognitive and MRI outcomes favored intervention	Attrition increased during extended follow-up and fewer participants contributed to 36-month efficacy analyses
Fortier et al., 2019 [54].	Randomized controlled trial; *n* = 52; participants with MCI.	30 g/day ketogenic MCT drink vs. placebo	6 months	Cerebral ketone/glucose metabolism and cognitive measures	Increased cerebral ketone utilization without restoring glucose metabolism; improvements in selected cognitive measures	Small sample, incomplete retention, and no evidence regarding conversion to dementia or disease modification
Krikorian et al., 2012 [56].	Randomized dietary intervention; *n* = 23; older adults with MCI.	Very-low-carbohydrate ketogenic diet vs. high-carbohydrate diet	6 weeks	Verbal memory and metabolic/ketone measures	Verbal memory improved with the ketogenic intervention and correlated with ketone concentrations	Very small sample and short duration
Henderson et al., 2020 [57].	Randomized placebo-controlled trial; *n* = 413; participants with mild-to-moderate AD.	AC-1204 ketogenic formulation vs. placebo	26 weeks	ADAS-Cog, global clinical status, functional outcomes	No significant cognitive, global, or functional efficacy in the prespecified primary population	Primary efficacy population focused on APOE ε4 non-carriers; findings did not confirm earlier genotype-specific signals
Phillips et al., 2021 [58].	Randomized crossover trial; *n* = 26; participants with clinically established AD.	Modified ketogenic diet vs. comparison low-fat/healthy diet	12 weeks per intervention	Cognition, activities of daily living, quality of life	Activities of daily living and quality of life improved, but cognition did not differ significantly	Small crossover sample and relatively short intervention
Buchholz et al., 2024 [59].	Randomized controlled feasibility trial; *n* = 38; older adults with MCI attributed to AD.	Modified Atkins diet vs. control diet	12 weeks	Memory, metabolic measures, feasibility and ketosis-defined adherence	No significant memory benefit; metabolic effects were observed	High attrition and very poor achievement of the prespecified ketosis-based adherence target

**Table 2:** Key Human Intervention Studies of B Vitamins and Vitamin D Relevant to Cognitive Impairment and Alzheimer’s Disease.

Study	Design, Population and Disease Stage	Intervention vs. Comparator	Duration	Main Outcomes	Main Findings	Relevant Limitations
Smith et al., 2010; de Jager et al., 2012 [83,84].	Randomized double-blind controlled trial; *n* = 271 adults >70 years with MCI; serial MRI available in a subset of *n* = 187.	Folic acid 0.8 mg/day + vitamin B12 0.5 mg/day + vitamin B6 20 mg/day vs. placebo	24 months	Whole-brain atrophy, cognition and clinical outcomes	B-vitamin treatment reduced the rate of brain atrophy; selected cognitive benefits were greatest in participants with higher baseline homocysteine	Single-center study; MRI available only in a subset; MCI not defined using contemporary amyloid/tau biomarkers
Kang et al., 2021 [101].	Randomized placebo-controlled VITAL ancillary studies; *n* = 3424 with telephone-based cognitive assessment and *n* = 794 with in-person assessment; generally healthy older adults.	Vitamin D3 2000 IU/day vs. placebo	≈2–3 years	Global cognition, verbal memory and executive function	No significant benefit on global cognitive decline or individual cognitive domains	Population was not enriched for cognitive impairment or marked vitamin D deficiency
Lönnroos et al., 2025 [102].	Randomized placebo-controlled trial; *n* = 2492; dementia-free older adults.	Vitamin D3 1600 IU/day or 3200 IU/day vs. placebo	Up to 5 years	Incident dementia and AD	No significant reduction in dementia or AD incidence	Few dementia events and generally adequate vitamin D status at baseline reduced statistical power to detect benefit
Corbett et al., 2025 [103].	Randomized placebo-controlled trial; *n* = 620 adults aged ≥50 years with mild-to-moderate vitamin D deficiency and early cognitive impairment.	Vitamin D3 vs. placebo	24 months	Executive function, global cognition, functional outcomes and well-being	No significant improvement in the primary executive-function outcome or secondary cognitive/functional outcomes	Early cognitive impairment was not equivalent to biomarker-confirmed AD; findings do not address severe vitamin D deficiency
Jia et al., 2019 [104].	Randomized double-blind placebo-controlled trial; *n* = 210; older adults with established AD.	Vitamin D 800 IU/day vs. placebo	12 months	Cognitive measures and peripheral Aβ-related biomarkers	Improvements were reported in selected cognitive outcomes and peripheral Aβ-related biomarkers	Single study; peripheral biomarkers do not establish modification of cerebral Aβ pathology and findings require replication

**Table 3:** Evidence Summary of Dietary Patterns and Micronutrient Interventions in Alzheimer’s Disease.

Key References	Dietary or Nutritional Strategy	Predominant Evidence Base	Main Take-Home Message	Key Limitations/Cautions
Nucci et al., 2024 [38]; Valls-Pedret et al., 2015 [43].	Mediterranean diet	Prospective cohorts, meta-analyses, randomized cognitive sub studies	Higher adherence is associated with better cognitive outcomes and lower dementia risk; randomized evidence is supportive but does not establish AD prevention or treatment.	Residual confounding; variable dietary definitions; cognition often secondary in intervention studies.
van Soest et al., 2024 [41]; Barnes et al., 2023 [44].	MIND diet	Mainly observational studies; one major 3-year randomized trial	Observational findings are favorable, but the largest randomized trial found no significant cognitive benefit over an active control.	Predominantly observational evidence; limited geographic diversity; active control may have reduced treatment contrast.
Tsai et al., 2026 [47]; Zhu et al., 2022 [105].	Healthful plant-based patterns	Prospective cohorts and meta-analyses	High-quality plant-based patterns are associated with better cognitive outcomes, whereas unhealthy plant-based patterns are not.	Evidence remains observational; plant-based indices do not necessarily represent vegetarian or vegan diets.
Katonova et al., 2022 [48]; Neufingerl & Eilander, 2022 [24]; Niklewicz et al., 2024 [50].	Strict vegan diet	Nutritional cohorts and reviews; very limited AD-specific intervention evidence	No evidence shows additional AD protection from complete exclusion of animal products.	Requires attention to B12, vitamin D, iodine, calcium, zinc, EPA/DHA, protein and energy adequacy.
Bohnen et al., 2023 [52]; Phillips et al., 2021 [58]; Buchholz et al., 2024 [59].	Ketogenic/modified ketogenic diets	Small randomized and crossover trials	Ketosis may improve brain energy availability and selected outcomes, but cognitive findings are inconsistent and disease modification is unproven.	Small samples, short duration, adherence difficulties, gastrointestinal effects, weight loss and possible lipid changes.
Fortier et al., 2019 [54]; Henderson et al., 2020 [57].	MCT/caprylic triglyceride formulations	Brain metabolic studies and randomized trials	Increase cerebral ketone utilization and may provide metabolic compensation, but consistent cognitive or functional benefit has not been established.	Heterogeneous formulations and responses; metabolic compensation does not imply modification of AD pathology.
Shah et al., 2013 [31]; Soininen et al., 2017 and 2021 [32,33].	Fortasyn Connect/Souvenaid	Randomized trials in prodromal and established AD	Some secondary and longer-term outcomes in prodromal AD are encouraging, but the primary 24-month cognitive endpoint was neutral and established AD trials have largely been negative.	Attrition, stage-dependent findings and lack of consistent clinically meaningful benefit.
Smith et al., 2010 [83]; de Jager et al., 2012 [84].	Vitamin B12, folate and B6	Observational studies, VITACOG and small AD trials	Treat deficiency and metabolic impairment; selected benefits may occur in MCI with elevated homocysteine, but routine supplementation is not an AD treatment.	Biomarker interpretation is complex; cognitive and functional benefits are inconsistent.
Zhang et al., 2024 [88]; Kang et al., 2021 [101]; Lönnroos et al., 2025 [102]; Corbett et al., 2025 [103].	Vitamin D	Cohorts, meta-analyses and randomized trials	Low vitamin D is associated with dementia risk, but randomized trials have generally not shown prevention or slowing of AD.	Reverse causality and confounding; variable thresholds; unnecessarily high doses may cause harm.

**Table 4:** Stage-Specific Clinical Application of Nutritional Care Across the Alzheimer’s Disease Continuum.

Key References	Clinical Stage or Context	Main Nutritional Goal	Clinically Reasonable Approach	Main Caution
Livingston et al., 2024 [12]; Yassine et al., 2022 [30].	Midlife and cognitively healthy aging	Support long-term cardiometabolic and cognitive resilience	Promote sustainable dietary quality and manage weight, blood pressure, glucose and lipids within a multidomain prevention strategy.	Do not present any specific diet, food or supplement as proven AD prevention.
Yassine et al., 2022 [30]; Samieri et al., 2022 [23,106].	Increased risk or preclinical AD	Preserve metabolic, vascular and nutritional reserve	Address poor dietary quality and clinically relevant deficiencies; integrate nutrition with broader risk-factor management.	Biomarker positivity does not establish that a particular diet will delay symptoms.
Volkert et al., 2024 [23]; Bohnen et al., 2023 [52].	MCI or early symptomatic AD	Prevent nutritional decline and correct reversible abnormalities	Monitor weight and intake, maintain adequate protein and energy, treat documented deficiencies and individualize experimental metabolic approaches.	Avoid unnecessary restriction, rapid weight loss and unsupported high-dose supplementation.
Volkert et al., 2024 [23]; Volkert et al., 2022 [109].	Moderate dementia	Preserve weight, muscle mass, hydration and safe oral intake	Individualize meals, energy/protein intake, hydration and feeding support; assess dysphagia when indicated.	Restrictive diets may worsen malnutrition and caregiver burden.
Volkert et al., 2024 [23].	Advanced or severe dementia	Prioritize comfort, dignity and safe feeding	Individualize oral feeding and symptom management according to goals of care and patient preferences.	Enteral feeding should not be used routinely in severe dementia or terminal care.
Volkert et al., 2024 [23]; Wolffenbuttel et al., 2024 [68]; Demay et al., 2024 [95].	Restricted diets/malabsorption/medication-related risk	Identify and correct specific deficiencies	Use targeted testing and replace documented deficiencies while monitoring nutritional adequacy.	Avoid indiscriminate testing or supplementation.
Waite et al., 2021 [51]; Volkert et al., 2022 [109].	Frailty, sarcopenia or unintended weight loss	Preserve muscle, function and independence	Increase energy and protein density and address reversible causes of poor intake.	Weight-loss or highly restrictive diets may be inappropriate even in individuals with obesity.

## Data Availability

No new data were created or analyzed in this study. Data sharing is not applicable to this article.
